# Supplementary material for: Combinatorial Effects of CPP-Modified Antimicrobial Peptides: Synergistic and Additive Interactions Against Pathogenic Bacteria
Source: Int J Mol Sci. 2025 Jun 21;26(13):5968. doi: 10.3390/ijms26135968 (PMC12250457; doi:10.3390/ijms26135968)
Supplement: Supplementary file 1 [file ijms-26-05968-s001.zip › ijms-3689121-supplementary.pdf]

Table S1. Characteristics of the peptides from chromatography and mass spectrometry analysis

1.	R23F^{S*}	RKKRRQRRRGG-Sar-GVVVHI-X-GGKF-NH2
	Retention time	14.6 min
	Peptide purity	>95%
	Calculated monoisotopic mass	2613.5541
	Observed monoisotopic mass (ESI MS)	2613.5240
2.	V31K^{S*}	G-VVVHINGGKFGG-Sar-GSRQIKIWFQNRR-X-KWKK-NH2
	Retention time	13.7 min
	Peptide purity	>95%
	Calculated monoisotopic mass	3663.1015
	Observed monoisotopic mass (MALDI MS)	3669.2
3.	R44K^{S*}	RKK-K-RQRRRGG-Sar-GVVVHINGGKFGG-Sar-GSRQIKIWFQNRR-X-KWKK-NH2
	Retention time	13.6 min
	Peptide purity	>90%
	Calculated monoisotopic mass	5141.0306
	Observed monoisotopic mass (ESI MS)	5141.0848

Project Name: testing_peptides
Reported by User: System

Breeze

SAMPLE INFORMATION

Sample Name:	Arg23Phe, 5	Acquired By:	System
Sample Type:	Unknow n	Date Acquired:	14.12.2020 11:32:01
Vial:	1	Acq. Method:	grad_3_2m_50_226_280
Injection #:	2	Date Processed:	14.12.2020 16:14:49
Injection Volume:	50,00 ul	Channel Name:	2487Channel 1
Run Time:	25,00 Minutes	Channel Desc.:	226 nm
Column Type:		Sample Set Name:	

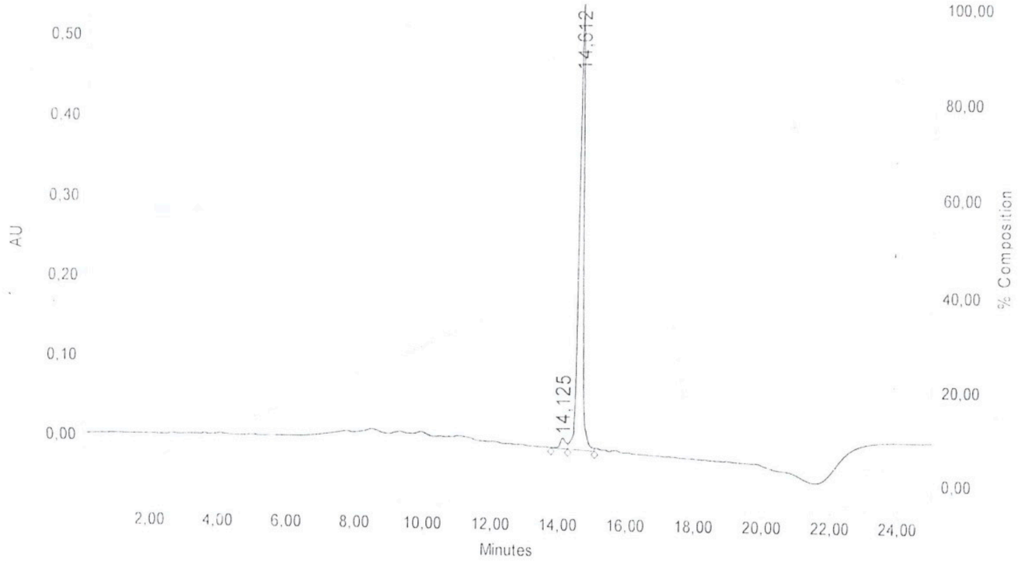

	RT (min)	Area (µV*sec)	% Area	Height (µV)	% Height
1	14,125	151635	2,86	13270	2,33
2	14,612	5143608	97,14	556721	97,67

Report Method: Gradient Overlay Report ASC   Printed 16:15:09   14.12.2020   Page: 1 of 1

Figure S1. Chromatogram of the peptide R23F[S*].

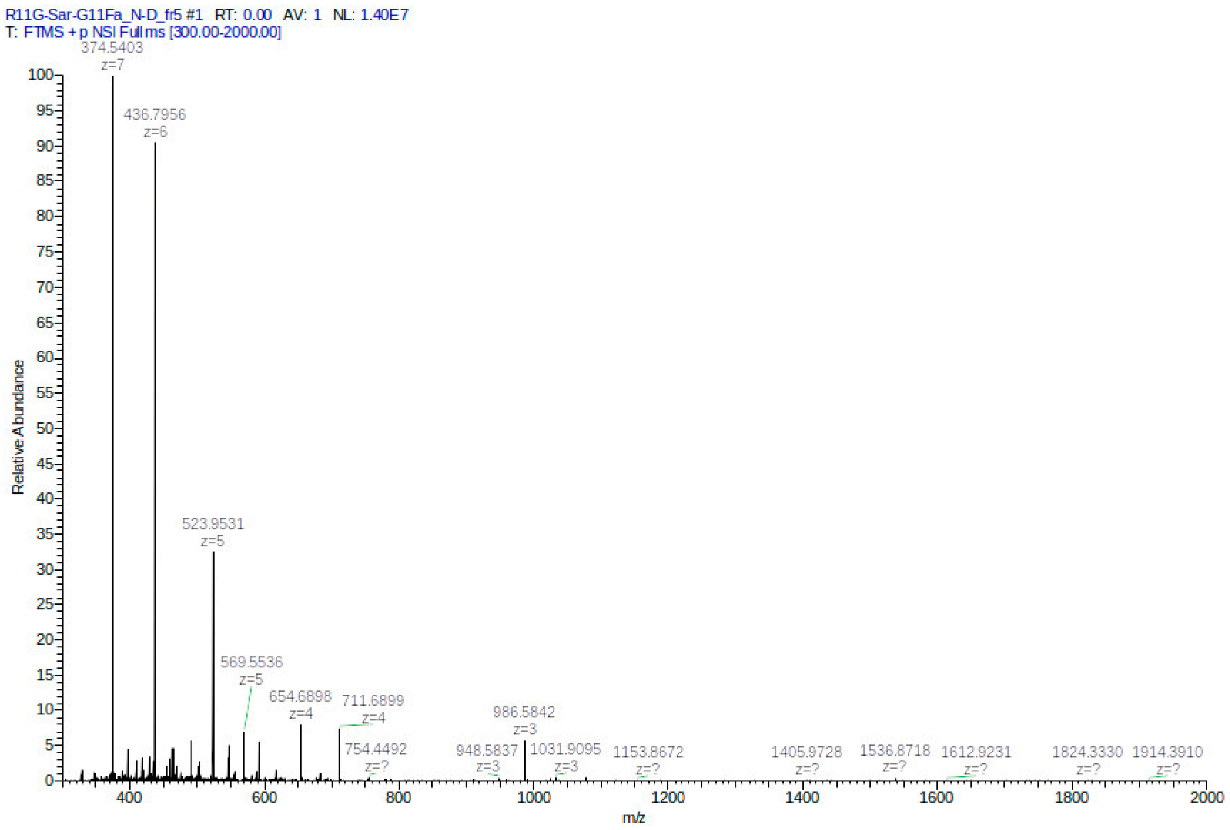

Figure S2. Mass spectrometry analysis for R23F^{S}*.

Project Name: testing_peptides
Reported by User: System

Breeze

SAMPLE INFORMATION

Sample Name:	G-VV13G-S17K (-Ac) short	Acquired By:	System
Sample Type:	Unknown	Date Acquired:	23.06.2022 12:02:00
Vial:	1	Acq. Method:	gradient1_5_80_25min
Injection #:	2	Date Processed:	23.06.2022 12:30:49
Injection Volume:	50,00 ul	Channel Name:	2487Channel 1
Run Time:	25,00 Minutes	Channel Desc.:	226
Column Type:		Sample Set Name:	

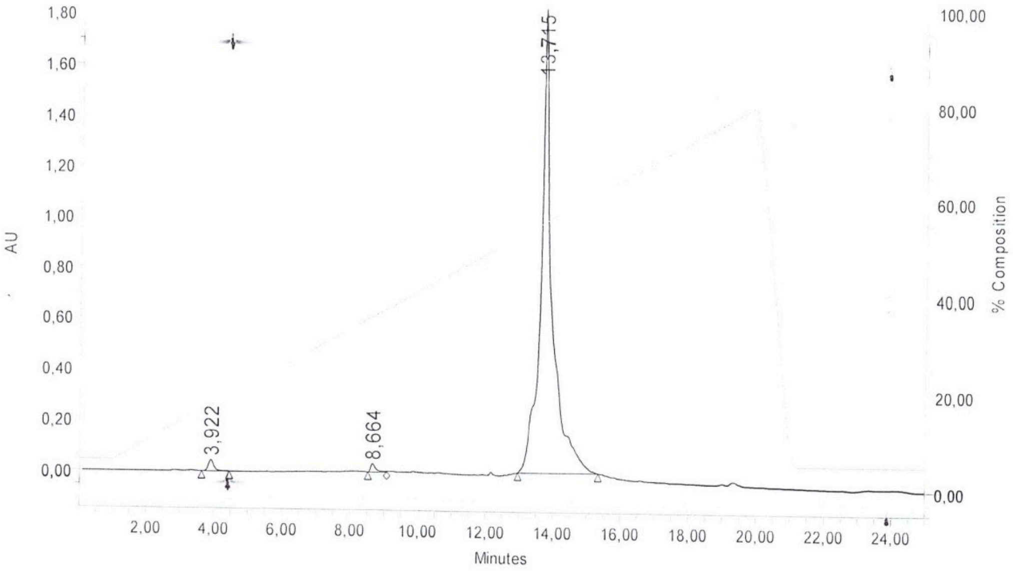

	RT (min)	Area (µV*sec)	% Area	Height (µV)	% Height
1	3,922	495075	1,15	43591	2,30
2	8,664	330876	0,77	31963	1,69
3	13,715	42222130	98,08	1817364	96,01

Report Method: Gradient Overlay Report ASC   Printed 12:54:42   23.06.2022   Page: 1 of 1

Figure S3. Chromatogram of the peptide V31K^{S}*.

2022-06-30

5/10

GVV13GS17K
plus ions

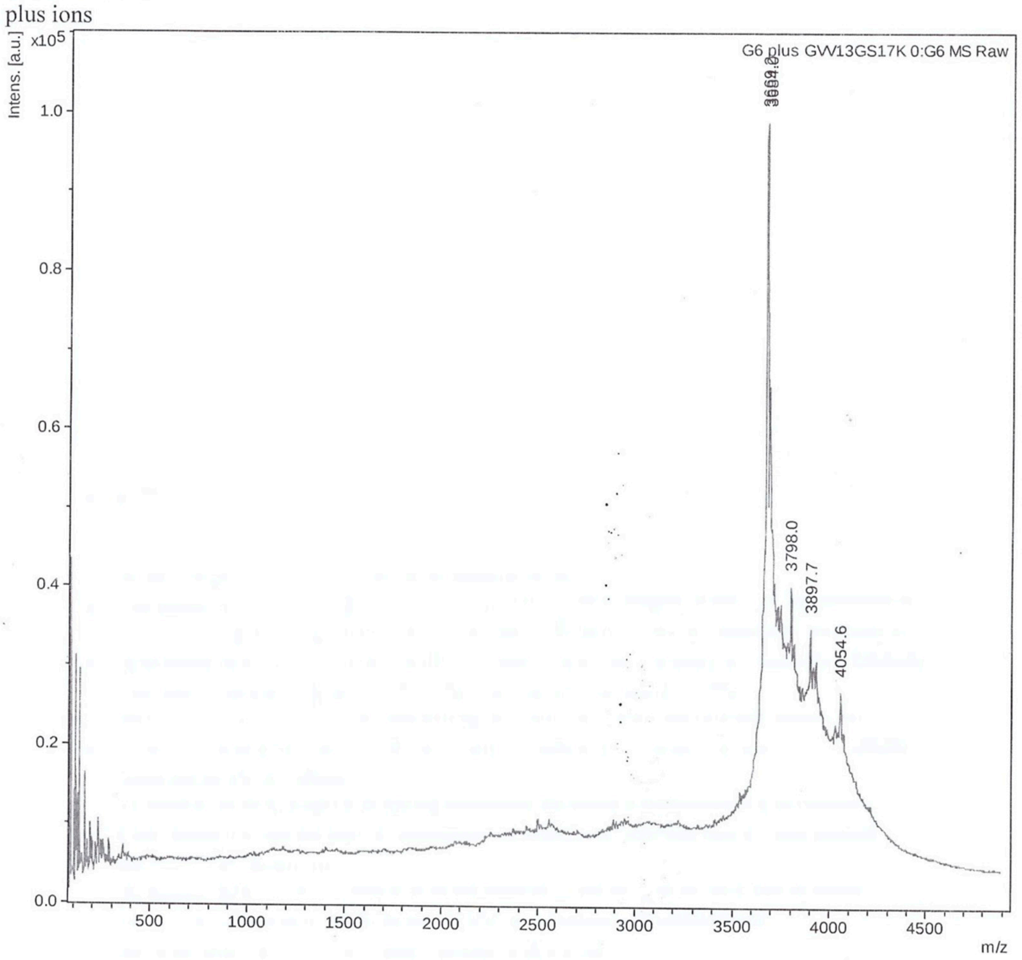

Figure S4. Mass spectrometry analysis for V31K^{S}*.

Project Name: testing_peptides
Reported by User: System

SAMPLE INFORMATION

Sample Name:	R13G-VVI3G-S17K, 10	Acquired By:	System
Sample Type:	Unknown	Date Acquired:	25.04.2022 12:58:26
Vial:	1	Acq. Method:	gradient1_5_80_25min
Injection #:	4	Date Processed:	25.04.2022 15:41:35
Injection Volume:	50,00 ul	Channel Name:	2487Channel 1
Run Time:	25,00 Minutes	Channel Desc.:	226
Column Type:		Sample Set Name:	

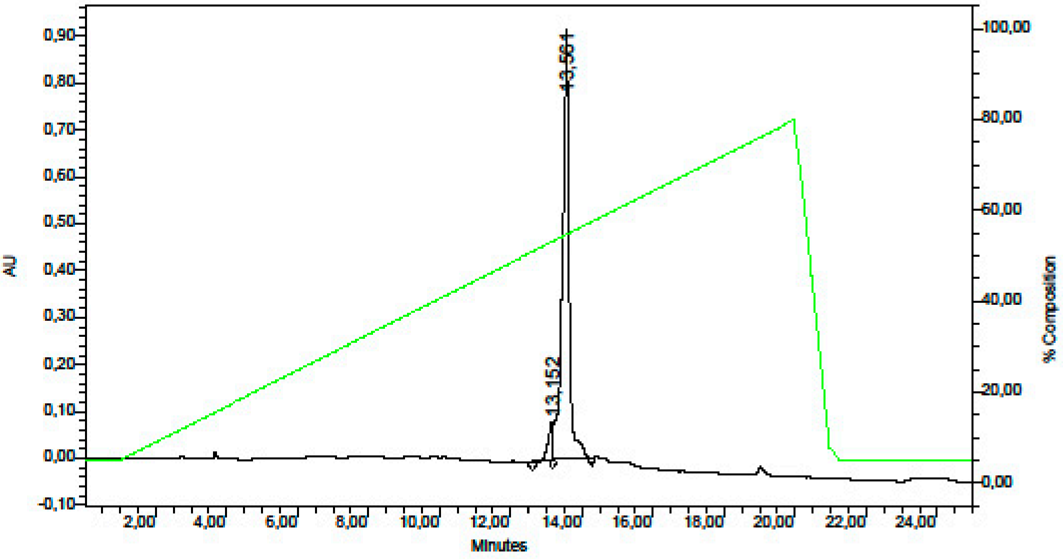

	RT (min)	Area (□V*sec)	% Area	Height (□V)	% Height
1	13,152	817763	6,38	84005	8,37
2	13,561	12000433	93,62	919754	91,63

Figure S5. Chromatogram of the peptide R44K^{S*}.

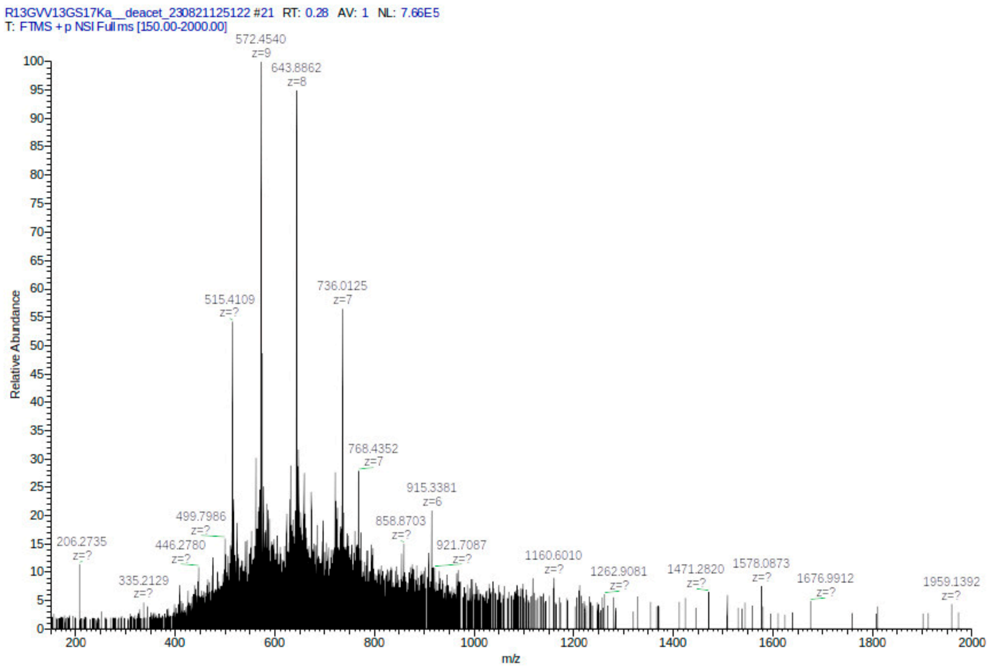

Figure S6. Mass spectrometry analysis for R44K^{S}*.
